# Quality of life during occlusion therapy for amblyopia from the perspective of the children and from that of their parents, as proxy

**DOI:** 10.1186/s12886-022-02342-w

**Published:** 2022-03-25

**Authors:** Geertje W. van der Sterre, Elizabeth S. van de Graaf, Helma M. van der Meulen-Schot, Ellen Abma-Bustraan, Henk Kelderman, Huibert J. Simonsz

**Affiliations:** 1 Department of Ophthalmology, Erasmus MC, University Medical Center Rotterdam, PO Box 2040, NL 3000 CA Rotterdam, The Netherlands; 2 Department of Ophthalmology, Reinier de Graaf Hospital Delft, PO Box 5011, NL 2600 GA Delft, The Netherlands; 3 Faculty of Social and Behavioural Sciences, University of Leiden, PO Box 9555, NL 2300 RB Leiden, The Netherlands

**Keywords:** Amblyopia, Occlusion therapy, Health-related quality of life, Health-related quality-of-life questionnaire, Visual acuity, Angle of strabismus

## Abstract

**Background:**

Parents pity their amblyopic child when they think that they suffer from occlusion therapy. We measured health-related quality of life during occlusion therapy.

**Methods:**

We developed the Amblyopia Parents and Children Occlusion Questionnaire (APCOQ). It was designed by a focus group of patients, orthoptists and ophthalmologists and consisted of twelve items concerning skin contact of patch, activities, contact with other children, emotions and awareness of necessity to patch.

Parents filled out the Proxy Version shortly before the Child Version was obtained from their child. Child Version item scores were compared with Proxy Version item scores and related to the child’s age, visual acuity, refraction, angle of strabismus, and cause of amblyopia.

**Results:**

63 children were recruited by orthoptists, and their parents agreed to participate. Three children were excluded: one child with Down-syndrome, one child with cerebral palsy, and one child who had been treated by occlusion therapy. Included were 60 children (mean age 4.57 ± 1.34 SD) and 56 parents. Children had occluded 128 ± 45 SD days at interview. Prior to occlusion, 54 children had worn glasses. Cronbach’s α was 0.74 for the Child Version and 0.76 for the Proxy Version. Children judged their quality of life better than their parents did, especially pertaining to skin contact and activities like games and watching TV. Notably, 13 children with initial visual acuity ≥ 0.6 logMAR in the amblyopic eye experienced little trouble with games during occlusion. Quality of life in eight children with strabismus of five years and older correlated negatively (Spearman rank mean rho = -0.43) with angle of strabismus. Children with amblyopia due to both refractive error and strabismus (*n* = 14) had, relatively, lowest quality of life, also according to their parents, as proxy. Several children did not know why they wore a patch, contrary to what their parents thought.

**Conclusions:**

Children’s quality of life during occlusion therapy is affected less than their parents think, especially regarding skin contact, playing games and watching TV during occlusion. Quality of life correlates negatively with the angle of strabismus in children five years and older. Children do not know why they wear a patch, contrary to what their parents think.

Notably, children with low visual acuity in the amblyopic eye, had little difficulty playing games.

## Introduction

Amblyopia (lazy eye) has a prevalence of approximately 3.25% [1]. It is defined by best-corrected visual acuity ≥ 0.3 logMAR in the affected eye with a 2 logMAR line difference between the two eyes and with the presence of an amblyogenic factor while there is no underlying structural abnormality of the eye. Amblyopia is treated before the age of eight years by glasses and by occluding the better eye for several hours per day with an occlusion patch. Patching the better eye for several hours may cause complaints of sweating, itching, pain and allergic reaction [2]. The removal of the patch might cause pain and redness around the eye patch [2]. Due to occluding the better eye, the patch could also prohibit daily functioning like writing, seeing on a schoolboard, colouring, drawing and painting, playing on the computer and perceiving obstacles [3]. At a social level the child might feel annoyed and be bullied by other children due to wearing a patch [4, 5]. These aspects may impair the child’s health-related quality of life (QoL) during occlusion therapy.

The child’s QoL during the occlusion therapy, in addition to the decrease in QoL due to the amblyopia itself, is critical for the cost-effectiveness of population-wide screening and treatment for amblyopia [6], because so many children get occlusion therapy. Hence, even a minor decrease in the child’s quality of life during the amblyopia treatment by occlusion can decrease the overall cost-effectiveness of amblyopia screening and treatment. For the calculation of the cost-effectiveness of screening and treatment of amblyopia, evidence has been collected regarding the effectiveness of the amblyopia screening [1], the costs of its treatment [7], the loss in quantified QoL from unilateral visual impairment caused by unsuccessfully treated amblyopia [8], the increased risk of bilateral visual impairment in unsuccessfully treated amblyopia patients [9] and the loss in quantified QoL when bilateral visual impairment occurs [10]. It has been suggested that the child’s quality of life may be decreased so much during occlusion therapy and so many children receive occlusion therapy that screening for amblyopia might not be cost-effective [6].

In this study, we measured the quality of life during occlusion therapy. We were especially interested in a possible discrepancy between the child’s perspective on its QoL during occlusion therapy and that of their parent [11, 12]. We developed a disease-treatment-specific QoL instrument: the Amblyopia Parents and Children Occlusion Questionnaire (APCOQ). Children were interviewed with its Child Version to obtain their QoL. The parents filled out the Proxy Version, as parallel, not substitute, respondents.

## Materials and methods

The APCOQ was, as our previously developed Amblyopia & Strabismus Questionnaire [13], developed by a focus group comprising two paediatric ophthalmologists, one orthoptist and one patient with residual childhood amblyopia. The development first concerned selecting the content of the questionnaire. We broadly followed the structure of the eleven themes that Carlton had developed for the CAT-QoL [3]. We began, late 2010, with the development of our questionnaire to assess QoL in children during occlusion therapy and their parents, as proxy. It was preceded by a study in 2005 from our research group where six questions were asked at children with occlusion therapy to evaluate their non-compliance, two of which concerned quality of life: “how did you feel when you wore the patch”, “what did other children say about your patch” [14]. Our first interview with a child and their parent was in March 2012, using the final APCOQ. In October 2011 Carlton published a discussion paper about the development of eleven themes for a child amblyopia treatment QoL instrument [3]. Its draft was online published in 2012; its themes had been reduced to eight after content analysis [15].

The topics that were adopted from Carlton [3] for the APCOQ were physical sensation of the patch on their eye, pain related to treatment, ability to undertake work at school and other things, feeling sad, being able to play with other children, treatment by other children. Finally, we included the last topic about the child’s awareness of the necessity to patch, which is not a quality of life issue, but about the implementation of the occlusion therapy [16].

The second phase in the APCOQ development was the operationalisation of the content into items and their basic phrasing. The requirements of operationalisation and phrasing were defined as: the functional implications of wearing the patch should apply to the daily life of all children, the psychological consequences should be understandable for all children and the items should be clear and distinctive for all children.

The third phase comprised of phrasing the Child Version items as negative questions, and the Proxy Version items as negative statements. The APCOQ, i.e. its Child Version and its Proxy Version, contains twelve items (Table 1).

**Table 1:** Amblyopia Parents and Children Occlusion Questionnaire

Items and answer categories of the APCOQ Child Version and the APCOQ Proxy Version
**Child Version**
1 Do you feel the patch on the eye? Not (5) A little (4) Moderate (3) Quite (2) Very (1)
2 Does the patch hurt or itches when it is on the eye? Not (5) A little (4) Moderate (3) Quite (2) Very (1)
3 Does it hurt when the patch is removed from the eye? Not (5) A little (4) Moderate (3) Quite (2) Very (1)
4 Are you able to undertake things at home or schoolwork when you wear the patch? Well (5) Rather well (4) Moderate (3) Rather bad (2) Bad (1)
5 How much troubled are you by the patch when playing on the computer; watching TV; colouring or making puzzles? Not (5) A little (4) Moderate (3) Quite (2) A lot (1)
6 Can you play with other children when you wear the patch? Well (5) Rather well (4) Moderate (3) Rather bad (2) Bad (1)
7 Are you sad when you have to wear the patch? Not (5) A little (4) Moderate (3) Quite (2) Very (1)
8 Are you angry when you have to wear the patch? Not (5) A little (4) Moderate (3) Quite (2) Very (1)
9 Are you cross with your mam or dad because you have to wear the patch? Not (5) A little (4) Moderate (3) Quite (2) Very (1)
10 Are you laughed at or bullied when you wear the patch? Not (5) A little (4) Moderate (3) Quite (2) Very (1)
11 Do you know why you have to wear the patch? Well (5) Rather well (4) Moderate (3) Rather bad (2) Bad (1)
12 Do you mention it to your mam or dad when they forget to put the patch on? Always (5) Mostly (4) Sometimes (3) Almost never (2) Never (1)
**Proxy Version (for the parents)**
1 My child feels the patch on the eye. Strongly agree (1) In general agree (2) Not certain (3) In general disagree (4) Strongly disagree (5)
2 The patch hurts or is itching my child when wearing it on the eye. Strongly agree (1) In general agree (2) Not certain (3) In general disagree (4) Strongly disagree (5)
3 The removal of the patch hurts my child. Strongly agree (1) In general agree (2) Not certain (3) In general disagree (4) Strongly disagree (5)
4 My child can undertake things at home or schoolwork when it wears the patch. Strongly agree (5) In general agree (4) Not certain (3) In general disagree (2) Strongly disagree (1)
5 My child has trouble from the patch when playing on the computer; watching TV; colouring or making puzzles. Not (5) A little (4) Moderate (3) Quite (2) A lot (1)
6 My child can play with other children when it wears the patch. Strongly agree (5) In general agree (4) Not certain (3) In general disagree (2) Strongly disagree (1)
7 My child is sad when it wears the patch. Strongly agree (1) In general agree (2) Not certain (3) In general disagree (4) Strongly disagree (5)
8 My child is angry when it wears the patch. Strongly agree (1) In general agree (2) Not certain (3) In general disagree (4) Strongly disagree (5)
9 My child is cross with us because it has to wear the patch. Strongly agree (1) In general agree (2) Not certain (3) In general disagree (4) Strongly disagree (5)
10 My child is laughed at or bullied when it wears the patch. Strongly agree (1) In general agree (2) Not certain (3) In general disagree (4) Strongly disagree (5)
11 My child knows why it has to wear the patch. Strongly agree (5) In general agree (4) Not certain (3) In general disagree (2) Strongly disagree (1)
12 My child mentions it to me when I forget to put the patch on. Strongly agree (5) In general agree (4) Not certain (3) In general disagree (2) Strongly disagree (1)

Item 1 is about whether the child could feel the patch on the eye; item 2 asks whether the patch hurts or itches when it is on the eye; item 3 concerns pain when the patch is removed [2]. These are the items that concern the child’s physical suffering from wearing the patch.

Item 4 asks about the child’s daily functioning at home, like putting things on the table or helping in the kitchen, or doing schoolwork like writing while wearing the patch. Item 5 asks about the troubles with undertaking activities or tasks while wearing the patch. The activities from which the child and their proxy had to choose the most troubling activity were: playing games on the computer, watching TV, colouring or making puzzles.

The next five items pertained to contact with other children and to emotions and were phrased as follows: playing with other children during occlusion therapy (item 6); being sad when patching (item 7); being angry when patching (item 8); being cross with parents because having to wear the patch (item 9); being teased or laughed at or bullied by peers when patching (item 10) [5].

Finally, item 11 and item 12 assessed the child’s awareness of the necessity to patch, which is important for a good compliance with occlusion therapy [16]. Item 11 asked whether the child knew why he has to wear the patch; item 12 whether the child mentioned it to its parents when they forgot to put the patch on.

APCOQ item answers were categorized on a five-point Likert scale ranging from score 1 to 5 (Table 1).

### Participants and APCOQ administration

Enrolled were three to seven year-old children who for the first time underwent occlusion therapy, could give reliable answers and could understand, as well as the accompanying parent, Dutch language. The child and their parents were recruited by orthoptists who treated the child from the Erasmus Medical Center Rotterdam (HMMS) and the Reinier de Graaf Hospital in Delft (HMMS, EAB) when they came to the ophthalmology outpatient clinic and either were first prescribed glasses and then diagnosed with amblyopia and prescribed occlusion therapy, or had immediately been diagnosed with amblyopia and were prescribed occlusion therapy.

Inclusion criteria were: newly diagnosed amblyopia worse than 0.3 LogMAR visual acuity in one eye (for 4 year old children 0.2) or at least 2 LogMAR lines difference in visual acuity between the two eyes, identified cause of amblyopia: deprivation, refractive error or strabismus and no other identifiable cause of decreased visual acuity.

The parents were contacted by telephone by ESG and the nature of the interview was explained to the parent. After informed consent was obtained from the child’s parent, we sent the Proxy Version of the questionnaire by postal mail to the parents, three weeks before the child-interview and it was filled out by them.

The twelve questions of the Child Version were read out by ESG to the child during a face-to-face interview at the ophthalmic outpatient clinic. The interview of approximately ten minutes was taken in the presence of the accompanying parent. It was held when the child came back at the first follow-up visit to the orthoptist. This follow-up was scheduled after three months of occlusion therapy, but in practice appointments were often made after four months or had to be postponed.

The total time spent with the child was not much more than the approximately ten minutes it took to read the questions to the child and record the responses. The interview with the child was done in a separate room, in the presence of a parent. The orthoptist would not be present during the interview. As much as possible the interview was scheduled before the first follow-up visit to the orthoptist.

The parent provided information about the location where the occlusion was carried out: at school, at home or at both places.

Data collection started in 2012 and concluded in 2019. It was a considerable effort to recruit 60 children who were newly starting with amblyopia treatment and their parents in a university clinic.

The study protocol followed the tenets of the Declaration of Helsinki.

### Occlusion and clinical assessment

The treating orthoptists noted the data concerning prescribed daily hours of patching; child’s age at start of occlusion and at date of interview. The interviewer assessed the fluency in the national, i.e. Dutch language of the accompanying parent, graded into five categories [17] : i) none; ii) poor; iii) moderate; iv) good; v) excellent.

Assessments of orthoptic examinations were taken from the child’s patient charts (reported by the treating orthoptist). If the child was first treated with glasses (in case of refractive amblyopia), visual acuity (VA) in logMAR of the better and worse eye was measured three times: at the start of treatment with glasses, at the start of occlusion therapy, and at the interview. If the child started immediately with occlusion therapy, VA was measured two times: at the start of occlusion therapy and at the interview. Interocular-acuity difference was calculated from the VA measurement at start of occlusion. Spherical refraction, cylindrical refraction, and axial refraction of both eyes were recorded to obtain their spherical equivalent and subsequently to determine anisohypermetropia.

The orthoptist reported cause of amblyopia: refractive error, strabismus or both refractive error and strabismus. They applied the following criteria: the presence of anisohypermetropia or astigmatism defined refractive error, the presence of a visible angle of strabismus or lack of unilateral central fixation (micro-strabismus) defined strabismus, and the presence of anisohypermetropia or astigmatism and a visible angle of strabismus or micro-strabismus defined both refractive error and strabismus. The orthoptists also specified the kind of strabismus and angle of strabismus.

### Statistical analysis

Internal consistency of the Child Version and of the Proxy Version was calculated by Cronbach’s α to assess their reliability. We calculated Pearson correlation r of the APCOQ item scores with anisohypermetropia, with angle of strabismus, and with occlusion regimen. Mean of Child Version and Proxy item scores were calculated from the quality of life APCOQ items, i.e. items 1–10. The items 11 and 12 were excluded from this calculation as these do not relate to quality of life. Mean of scores groups categorized according to causes of amblyopia, according to the locations where patching was carried out, and according to the categories of fluency in the national language were calculated.

## Results

### Participants

Sixty children (22 female, 38 male) were included in the study: the APCOQ Child Version was obtained from them. One child with Down-syndrome, one with cerebral palsy and one child who occluded for several years already were excluded. Parents of 56 children (47 mothers, 9 fathers) filled out the Proxy Version as four parents did not sent it back, three of which had moderate fluency in the national language. Children were, on average, at start of occlusion therapy 4.57 year old (± 1.34) with a range of 2.6 to 8 years. 16 Children fell into age category of 2 – 4 years, 18 into age category of > 4 – 5 years, 11 into age category of > 5 – 6 years, and 15 into age category of > 6 years.

### Internal consistency of APCOQ

Before analysis of internal consistency, missing scores (in the Child Version 7 out of 720 answers, in the Proxy Version 17 out of 672 answers) were imputed by per item average scores. Cronbach’s α of the Child Version was 0.74 and of the Proxy Version 0.76.

### Child Version scores vs. Proxy Version scores

**Fig. 1:**
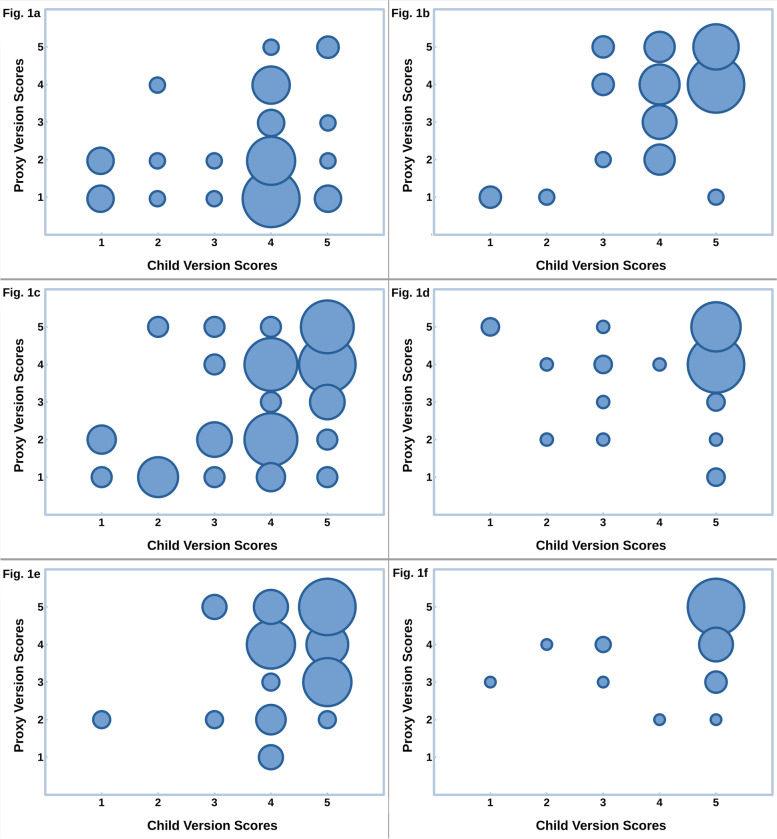

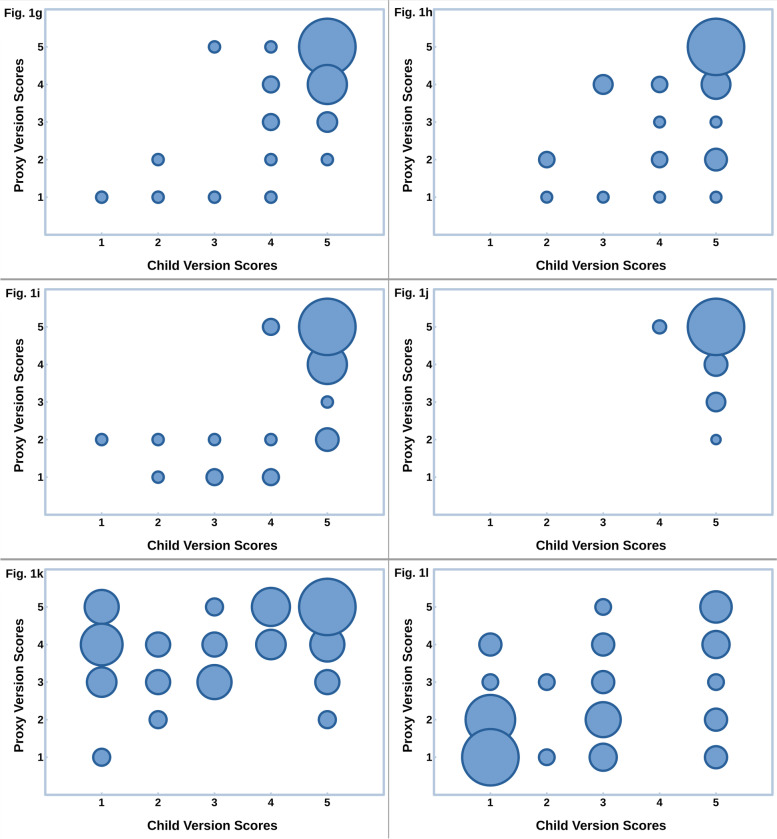
APCOQ Child and Parent Version frequencies of scores The frequencies of scores on the twelve APCOQ items from the Child Version are represented on the abscissa, and from the Proxy Version on the ordinate. The size of the bubble reflects the number of children and parents with that combination of scores on the item. In items 1 to 10, scores range from 1, lowest quality of life to 5, highest quality of life. In items 11 and 12, scores range from 1, lowest awareness of necessity to patch to 5, highest patch-necessity awareness. The charts are headed by the item number and the title; some titles are abbreviated for conciseness

Bubble graphs (Fig. 1) show the combined frequencies of the Child Version and Proxy Version scores, ranging from 1 (lowest quality of life) to 5 (highest quality of life) on items 1–10, and 1 (lowest patching awareness) to 5 (highest patching awareness) on items 11, and 12.

For most of the children, feeling the patch on the skin was not so troubling, but most parents thought it was (Fig. 1a). For the children, pain or itching of the patch was little troubling, and the parents, as proxy, thought the same (Fig. 1b). On removal of the patch, parents thought that the children suffered more than the children experienced it themselves (Fig. 1c).

Children had little trouble to undertake things at home or doing schoolwork when wearing the patch (item 4), and the parents agreed (Fig. 1d). Children were hardly troubled by the patch when playing games on the computer, watching TV or colouring (item 5), whereas parents thought that they were really troubled (Fig. 1e).

Almost all children (54), could play well with other children when wearing the patch (item 6). Some parents thought they could play less well than the children thought themselves (Fig. 1f). Almost all children (57) were not laughed at or bullied by other children when wearing the patch (item 10), and almost all parents agreed (Fig. 1j).

Almost no children were sad when wearing the patch (item 7) whereas some parents thought their child was a little sad (Fig. 1g). Almost no children were angry when wearing the patch (item 8) whereas some parents thought that their child was a little angry (Fig. 1h). Almost no children were cross with their parents because of wearing the patch (item 9), whereas some parents thought that their child was slightly cross. (Fig. 1i).

Several children did not know why they had to wear the patch (item 11), contrary to what their parents assumed that their child knew (Fig. 1k). Most children did not mention it to their parents when they forgot to patch (item 12), and this was confirmed by their parents, as proxy (Fig. 1l). Some parents interrupted regarding the child’s awareness of the necessity to patch, after the child’s answer had already been scored.

### Associations with demographic and occlusion data

We were unable to demonstrate a correlation between the child’s age at interview and the Child Version item scores and Proxy Version item scores, nor could we find a correlation between the occlusion regimen and item scores. Little difference was found between the mean scores from the three groups categorized according to location where occlusion was carried out and between the mean scores from the two groups categorized according to the parent’s fluency of Dutch language (Table 2).

**Table 2:** Association of item scores with occlusion data

Location of carrying out occlusion therapy		
	Item scores	
Location	Child Version	Proxy Version
Home mean ± SD	4.43 ± 0.26	3.69 ± 0.43
School mean ± SD	4.26 ± 0.54	3.99 ± 0.65
Home/School mean ± SD	4.34 ± 0.27	3.78 ± 0.74
Parents’ fluency as native Dutch		
	Item scores	
Category fluency	Child Version	Proxy Version
Good and less mean ± SD	3.94 ± 0.38	2.91 ± 0.85
Excellent mean ± SD	4.48 ± 0.28	3.89 ± 0.46

### Associations with clinical data

We tested the association of the APCOQ Child Version item scores and those of the APCOQ Proxy Version with the child’s visual acuity, with improvement of visual acuity and with angle of strabismus.

We found no correlation between the item scores of the Child Version and those of the Proxy Version and visual acuity of the worse eye at start of occlusion. Significantly, it was hardly troubling for the thirteen children with visual acuity at start of occlusion of ≥ 0.6 logMAR in the amblyopic eye to play games on the computer, to watch TV or to colour and to make puzzles. Visual acuity at start of glasses, at start of occlusion therapy and at interview, i.e. the improvement in VA, is presented in Table 3.

**Table 3:** Improvement in children’s visual acuity

VA (mean) at start glasses, start occlusion and at interview			
	Glasses	Occlusion	Interview
VA (logMAR) better eye	0.17 ± 0.17	0.05 ± 0.10	0.03 ± 0.12
VA (logMAR) worse eye	0.55 ± 0.29	0.41 ± 0.32	0.17 ± 0.22

We found no correlation between interocular-acuity difference at start of occlusion therapy and item scores.

Finally, we could not find a correlation between anisohypermetropia and item scores of the Child Version and the Proxy Version.

Twenty-three out of 60 children had strabismus. It was, in 21 out of the 23 children, caused by partial accommodative esotropia, in one case by intermittent exotropia and in one case by congenital fibrosis of extra-ocular muscles. Angle of strabismus (range > 0°—15°) and item scores of all children of five years and older (*n* = 28) correlated to some extent with the Child Version scores (Spearman rank mean rho = -0.22). However, in children of five years and older with strabismus (*n* = 8), strabismus angle correlated more strongly with the Child Version scores (Spearman rank mean rho = -0.43).

Children with amblyopia caused by both refractive error and strabismus had the lowest quality of life, as compared to those with amblyopia caused by refractive error and to those with amblyopia caused strabismus. This was confirmed by the parents, as proxy. Table 4 showed results from the three groups categorized according to cause of amblyopia. The 14 children who had both refractive error and strabismus also had the lowest visual acuity in the worse eye: mean logMAR 0.58 ± 0.25, against mean logMAR 0.47 ± 0.28 in the nine children with strabismus, and mean logMAR 0.33 ± 0.22 in the 37 children with refractive error, as cause of strabismus.

**Table 4:** Influence of amblyopia causes on item scores (mean) and VA (mean)

Amblyopia cause			
Results	Refractive error	Strabismus	Refr + Strab
Number of children	37	9	14
Child Version mean ± SD	4.4 ± 0.37	4.59 ± 0.41	4.14 ± 0.28
Proxy Version mean ± SD	3.97 ± 0.49	4.04 ± 0.61	3.39 ± 0.43
Visual acuity logMAR mean ± SD	0.33 ± 0.22	0.47 ± 0.28	0.58 ± 0.25

## Discussion

Children’s quality of life was affected less by occlusion therapy than their parents, as proxy, thought. Parents are quick to pity their child when the better eye is patched. According to the children, QoL was affected little by feeling the patch on the eye, or pain when the patch was removed. They were little trouble when playing games on the computer, watching TV, colouring or making puzzles.

Quality of life during occlusion was affected little by low initial visual acuity in the amblyopic eye: the 13 children with VA ≥ 0.6 logMAR in the amblyopic eye at the start of occlusion had no trouble with playing games on the computer or colouring or making puzzles. However, the visual acuity of the worse eye had improved between start of occlusion and interview by, on average, 0.24 logMAR. Hence, it can be argued that the children made a judgement about their current QoL on the basis of the improved visual acuity, whereas the parents remembered the difficulty with starting the occlusion a few months before.

Interestingly, when we measured quality of life with the Amblyopia & Strabismus Questionnaire [18] in 35-year olds with amblyopia who had been treated 30 years previously, we found a profound influence of the visual acuity of the amblyopic eye on quality of life. Blurred vision is serious for a 35-year-old adult as it affects daily-life functioning, like driving a car, and he or she is conscious of the fact that visual acuity of the worse eye will never improve.

It has been suggested [19] that parents can discern observable pain, e.g. pain uttered by the child, better than non-observable behaviour, like emotion. Our study results are partly compatible with that suggestion. Children and parents, as proxy, largely agreed in their report about non-observable emotions of anger and awareness of the necessity to patch. They disagreed in their report on pain or itching during skin contact of the patch and trouble with performing eye-or-hand tasks. It is possible that the children presented themselves as more brave by underreporting the pain or trouble in eye-or-hand tasks.

It was remarkable that the angle of strabismus correlated significantly with the APCOQ item scores in children of five years and older with strabismus (*n* = 8) (Spearman rank mean rho = -0.43).

Children with amblyopia caused by both refractive error and strabismus had, relatively, the lowest quality of life, also according to their parents; they also had the lowest visual acuity in the worse eye.

A limitation of this study is that visual acuity had already improved by wearing glasses before the start of the occlusion therapy (Table 3) (*n* = 45). However, the increase in visual acuity of the worse eye by glasses (average 0.14 logMAR) was less than the increase in visual acuity during the subsequent occlusion therapy (average 0.24 logMAR).

Another limitation is the frequency of high scores on item 10 (Fig. 1j) of both the APCOQ Child Version and the APCOQ Proxy Version was high, implying that a ceiling effect had occurred. A similar effect occurred in the CAT-QoL with the item of being laughed at or bullied by other children [20]. On the one hand it meant that this item was not appropriate to measure this aspect of quality of life, on the other hand it meant that bullying and being laughed at almost never occur in young children patched for amblyopia.

## Data Availability

The de-identified datasets generated and analysed during the current study are available in the Open Science Framework repository, https://osf.io/9qvn6/?view_only=ee19a502c1af4dac81944a618e34d10a
