# Correction to: Quality of life during occlusion therapy for amblyopia from the perspective of the children and from that of their parents, as proxy

**DOI:** 10.1186/s12886-022-02383-1

**Published:** 2022-04-21

**Authors:** Geertje W. van der Sterre, Elizabeth S. van de Graaf, Helma M. van der Meulen-Schot, Ellen Abma-Bustraan, Henk Kelderman, Huibert J. Simonsz

**Affiliations:** 1 Department of Ophthalmology, Erasmus MC, University Medical Center Rotterdam, PO Box 2040, NL, 3000 CA Rotterdam, The Netherlands; 2 Depart- ment of Ophthalmology, Reinier de Graaf Hospital Delft, PO Box 5011, NL, 2600 GA Delft, The Netherlands; 3 Faculty of Social and Behavioural Sciences, University of Leiden, PO Box 9555, NL, 2300 RB Leiden, The Netherlands

**Correction to: BMC Ophthalmol 22, 135 (2022)**

**https://doi.org/10.1186/s12886-022-02342-w**

Following the publication of the original article [1], we were notified that due to a misunderstanding during the proofing process, Figure 1 was not displayed on two separate pages, the way that the author originally intended.

The original article has been corrected.
